# Dermatology

**Published:** 1894-09

**Authors:** Henry Waldo


### DERMATOLOGY.

Dr. Unna, of Hamburg, was present at the Dermatological section of the British Medical Association at Bristol, and in very good English gave details of many of his more recent investigations, to some of which reference is made on page 203. He demonstrated sections of drawings, illustrating the fat-forming functions of the sweat glands in health and disease.

An assistant of his (Dr. Menahem Hodara) when living at Constantinople noticed that trichorrhexis was endemic in ladies' hair. This disease is exceedingly rare at Hamburg; but from a cultivation of the bacillus from Constantinople the discoverer was able to convey the disease to a patient at Hamburg.

At the Bristol meeting a most interesting paper by Dr. R. L. Bowles on "The Influence of Solar Rays on the Skin" was summarised in the following way:—(1) That heat *quâ* heat is not the cause of sunburn. (2) That there is strong evidence for believing that it is caused by the violet or ultra-violet rays of light reflected from the snow, which reflected light is not necessarily of the same quality as that which is incident. (3) Captain Abney finds that the violet or ultra-violet rays are very strong at high altitudes, and believes that altitude has much to do with sunburn. (4) That altitude alone does not explain sunburn; for one may not be sunburnt on rocks, say at 10,000 feet, and yet be immediately affected on descending to a glacier 3,000 or 4,000 feet lower down. (5) That sunburn and snow-blindness arise from similar causes, and that sunstroke may be associated with them. (6) That rays from the electric light produce much the same results as sunrays reflected from snow. (7) That the bronzing of the skin and the browning of the wooden châlets are probably produced by rays reflected from snow.

Skin diseases have received a large amount of attention of late, not only in the medical periodicals, but also at a Dermatological Congress recently held in London to inaugurate the new Dermatological Society of Great Britain and Ireland. At this congress, which lasted over four days, there were collected together at the different meetings a large number of patients exhibiting various affections of the skin. Among them were three or four cases which no one could name. One of these was a papular eruption upon a young woman's neck, which much resembled adenoma sebaceum; but Dr. Abraham, who exhibited the patient, had examined a papule and found on section an inflammatory neoplasm and no glandular hypertrophy or "adenomatous" structure. The papules were discrete and about the size of millet seeds. Dr. Eddowes showed a patient with Bazin's disease, illustrating "stocking" symmetry. This is described by Jonathan Hutchinson[1] as Bazin's malady—*érythème indurée des scrofuleux* or colloquially "Bazin's legs." Mr. Hutchinson says: "Under this name we recognise cases in which indurations form subcutaneously (lymphatic trunks probably) on the legs between knee and ankle, which are attended in the first stage by dusky erythema of the overlying skin, and in the second by unhealthy ulcerations, with undermined edges. Bazin himself did not describe the ulcerations, and named the disease '*érythème indurée des scrofuleux*'; but as the ulcers are by far the most conspicuous and most important feature, being the one almost certain to lead to an erroneous suspicion of syphilis, it is, I think, better to disuse his expression as too limited. 'Bazin's legs' derive their peculiarity no doubt from the part affected, are exposed somewhat to cold, and at some disadvantage in reference to the forces of the circulation. Its subjects are usually somewhat weakly, and often distinctly scrofulous, if not tuberculous. Now and then other parts than the legs are affected, the lower parts of thighs, the backs of upper arms or the ears, and sometimes the patients are liable to pustular ophthalmia. The ulcers are difficult to treat, and leave conspicuous scars with pigmented edges. Although most common in youth, the disease is not by any means restricted to it. Indurations precisely similar to those described occur very frequently in secondary syphilis, and now and then in diabetes. The syphilitic form can, indeed, in some cases be distinguished only from the scrofulous form by the history. In all the portraits illustrating Bazin's malady which are exhibited in the museum

[1] *Arch. Surg.*, July, 1894.

the history was carefully taken, and all suspicion of syphilis excluded."

Dr. Byrom Bramwell[1] says, that having satisfied himself that thyroid extract, when given by the mouth, undoubtedly exerts a powerful influence upon the nutrition of the skin, and that it is of real use in psoriasis and probably in some other forms of skin disease as well, he is desirous of satisfying others. He was led to try the effect of thyroid extract in diseases of the skin, not as a mere chance experiment, but with a definite therapeutic purpose. The extraordinary improvement in the nutrition of the skin, accompanied as it is in many cases with considerable desquamation, which the thyroid treatment produces within the course of a few weeks in cases of myxœdema and sporadic cretinism, suggested the idea that the remedy would probably prove useful in some skin diseases. In cases of myxœdema, the firm œdema of the subcutaneous tissues rapidly disappears under the administration of thyroid extract. The harsh, rough, dry skin soon becomes smooth, soft and moist. The sweat secretion, which was formerly entirely arrested, quickly becomes re-established. The superficial layers of the cuticle are detached, the desquamation in some cases being as profuse as in severe cases of scarlet fever. The dirty encrusted scalp becomes clean and smooth; the harsh, dry, ragged hair becomes soft and silky, and a profuse growth of new hair, which even in old people is usually well pigmented, is in the course of a few months produced. It is hardly an exaggeration to say that myxœdematous patients get a new skin after a course of thyroid treatment.

Dr. Hermann Klotz, writing on the principles of antisepsis in the treatment of eczema, says[2] that in dermatology, although the object of its study and its therapeutic actions, the outer integument of the human body, is constantly and in its entirety exposed to all the dangers of microbic infection, comparatively little has been written or said of antisepsis.

Experience teaches that the intact corneous layer of the epidermis furnishes ample protection against the entrance through the skin of the pathogenic microbes into the lymph and blood circulation, but that the slightest break in its continuity opens a door to infection, not only with the virulent elements of suppuration and sepsis, but also with those of syphilis, tuberculosis, &c. It is evident that many cases of eczema afford the most favourable opportunity for the many microbes with which our atmosphere is charged, the more so the more we are surrounded by the blessings of civilisation, to settle down, grow on the serous secretion, which furnishes a splendid culture medium, to enter the lymph channels and through them the blood-vessels,

[1] *Brit. J. Dermat.*, vol. vi., 1894, p. 193.

[2] *J. Cutan. & Genito-Urin. Dis.*, vol. xii., 1894, p 99.

causing local or general blood poisoning. In fact, it has been shown that microbes, pathogenic and apparently non-pathogenic, are constantly found in large numbers and varieties on the skin, so that it seems almost impossible that a portion of the surface deprived of the cover of the epidermis would remain uninfected even for a moment. With our present knowledge we must assume that an infection with pus-forming microbes has taken place, that conditions similar to those of a suppurating wound or of a suppurating mucous membrane are present. Dr. Klotz is inclined to believe, with Pick,[1] that suppuration, the so-called stage of eczema impetiginosum, makes its appearance when local septic infection complicates the moist stage. Where the exudation on the surface is not present, or in so moderate a degree that no moist surface is presented (eczema erythematosum, papulosum et squamosum), the excoriations resulting from scratching afford an equally commodious entrance to all kind of septic material, and in their turn often actually become the seat of more or less deeply seated suppuration. As long as suppuration exists, a cure is apparently out of the question. This will not be denied even by those who consider eczema as almost always a constitutional disease, produced by some irregularities in the functions of some organ or in the general household of the human body, and who, as a rule, look to internal or constitutional treatment as the most important part of therapeutics. Dr. Klotz says he does not hesitate to declare that carbolic acid is but rarely beneficial in diseases of the skin. Other drugs have taken the place of carbolic acid in antiseptic surgery; but almost invariably has the effect on the skin been equally disastrous, particularly with corrosive sublimate and iodoform. It seems not improbable that this incongruity of the most popular antiseptic remedies with the skin in its healthy state, as well as in pathological conditions, has been one of the principal factors to prevent the introduction of antiseptic principles into the treatment of eczema. Another cause, Dr. Klotz believes, has been the almost universally accepted doctrine which forbids the use of water in the management of eczema. To obtain favourable results it would be advisable to begin treatment by careful cleansing, and to apply the antiseptic remedies in the form of solutions instead of the usual ointments, as it is a well-known fact that the antiseptic drugs act much more powerfully in solutions than in combination with oils, grease, and other constituents of ointments. Whenever an impetiginous eczema presents itself for treatment, the first task, Dr. Klotz says, is to disinfect the diseased surface by removing the crusts and all other encumbrances with water and soap, Castile soap being generally preferred; and afterwards with a solution of corrosive sublimate, not stronger than one part in 3,000 or 5,000 parts of water. Thereafter the patients are advised to wash the

[1] *Prag. med. Wchnschr.*, 1883, p. 13.

diseased portions with this solution morning and evening by means of a pad of absorbent cotton, or a piece of lint or clean cloth (never with a sponge). In the evening, and if possible in the morning, lint or cotton pads, thoroughly soaked with the solution, are to be applied for one-half to one hour, during which time the pads have to be kept constantly moist. Then the parts are carefully dried, and a mild ointment, generally a ten per cent. boric acid vaseline, is rubbed into the skin and whenever feasible covered with lint or cotton and a bandage. This treatment is to be continued until a clean surface is presented on the affected parts and all suppuration has ceased, a result mostly obtained in a few days. In children, or in milder cases in grown patients, a two to three per cent. solution of boric acid takes the place of the corrosive sublimate, combined with the boric acid vaseline. Infants get their daily bath as usual; on removal from the water they are washed with the solution of boric acid. Circumscribed patches of eczema, particularly those showing the most aggravated conditions, are kept more or less constantly covered with cotton or lint compresses moistened with the solution, or rubbed and covered with the ointment. The results in such cases have been very satisfactory, and the moist stage of the eczema has been considerably shortened. In cases of very acute inflammation, particularly in eczema of the legs, with or without ulcers, two to four parts of acetate of lead are added to each 1000 parts of the boric acid solution, and compresses soaked with it are constantly applied for several days. This combination of boric acid and acetate of lead has recently been recommended by Saalfeld. Patients may be ordered fifteen to twenty grammes of the boric acid, and two to five grammes of acetate of lead, which they are directed to dissolve in one quart of boiled water while hot. In private practice a small quantity of glycerine and alcohol may be added: the substitution of about one-third or one-quarter of the water by lime water is of some advantage; it causes the precipitation of the insoluble portions in the shape of a very fine film. The same applications are made in all cases apparently not infected with purulent substances, where a moist surface exists, either circumscribed with the shape of the original vesicles preserved, or on larger patches. They are continued with as little interruption as possible until the discharge becomes less and the epidermis is rapidly forming a new protecting cover. Even in the dry forms of eczema, particularly in the erythematous ones, the wet applications and the washing with the antiseptic solutions are of great value. Boric acid is the one remedy which is well borne under any circumstances and on all localities; but under the principles of antisepsis, solutions of other drugs may be substituted, and will produce equally good effects. Thus ichthyol, one or two parts in 1000; salicylic acid, resorcin, thymol, permanganate of potassium, tannin, acetate of aluminia, even carbolic acid, may be quite as valuable if properly diluted.

Dr. Klotz says almost all the modern remedies owe their claims to their antiseptic action, and that their usefulness will increase in proportion with their deleterious effects on pathogenic microbes on the one side, and their non-irritating character towards the skin on the other. If we compare the different methods of treatment advocated by different dermatologists, it will not be difficult to discover in every one distinct traces of antiseptic principles.

HENRY WALDO.

